# Multiagent Systems Based Modeling and Implementation of Dynamic Energy Management of Smart Microgrid Using MACSimJX

**DOI:** 10.1155/2016/9858101

**Published:** 2016-04-04

**Authors:** Leo Raju, R. S. Milton, Senthilkumaran Mahadevan

**Affiliations:** SSN College of Engineering, Chennai, Tamil Nadu 603110, India

## Abstract

The objective of this paper is implementation of multiagent system (MAS) for the advanced distributed energy management and demand side management of a solar microgrid. Initially, Java agent development environment (JADE) frame work is used to implement MAS based dynamic energy management of solar microgrid. Due to unstable nature of MATLAB, when dealing with multithreading environment, MAS operating in JADE is linked with the MATLAB using a middle ware called Multiagent Control Using Simulink with Jade Extension (MACSimJX). MACSimJX allows the solar microgrid components designed with MATLAB to be controlled by the corresponding agents of MAS. The microgrid environment variables are captured through sensors and given to agents through MATLAB/Simulink and after the agent operations in JADE, the results are given to the actuators through MATLAB for the implementation of dynamic operation in solar microgrid. MAS operating in JADE maximizes operational efficiency of solar microgrid by decentralized approach and increase in runtime efficiency due to JADE. Autonomous demand side management is implemented for optimizing the power exchange between main grid and microgrid with intermittent nature of solar power, randomness of load, and variation of noncritical load and grid price. These dynamics are considered for every time step and complex environment simulation is designed to emulate the distributed microgrid operations and evaluate the impact of agent operations.

## 1. Introduction

Industrial automation is reaching a new level due to initiative taken towards industry 4.0. Conventional engineering approaches require every future interaction to be known in design time, whereas the future approaches require adaptive systems capable of efficiently adapting to failures, component replacements, and changes in the environment with less human intervention. They should be scalable, adaptable to unforeseen situations, and capable of handling evolution of their complexity [1]. These characteristics can be realized by enabling the system to dynamically reorganize by itself. These types of systems are getting more complex, open, and critical because they are adopted to remotely supervise and control most critical infrastructure utilities such as power grids and water transportation. MAS has received compelling attention in the recent times because of its capability of significantly improving the operational efficiency, reducing cost, and increasing degree of operational redundancy.

The world today is witnessing a gradual transition in the form of generation and distribution of electricity. The liberalization of the energy industry, together with decarbonization and inclusion of renewable energy sources, as well as ground-breaking Information and Communication Technologies (ICT) developments, has huge consequences on the way the sector works: we are moving rapidly towards a more decentralized, more sustainable, and smarter power system. The smart grid paradigm represents a transition towards intelligent, digitally enhanced, two-way power delivery grids [2]. Microgrid is the building block of smart grid and is poised to play a major role in enabling the widespread adoption of renewable, distributed energy resources.

However, renewable energy integration into the microgrid entails new challenges for control as the power generated is intermittent in nature, which impacts the dynamics and stability of the microgrid [3]. To meet these challenges, microgrid monitoring should incorporate communication and control to achieve an optimal balance between generation, energy storage, and load demand and also react quickly to changes. Dynamic energy management is a key enabler for the integration of renewable power generation onto the microgrid. Smart energy management of microgrids using genetic algorithm and neural networks is discussed in [4]. Energy management of hybrid renewable energy generation using fuzzy logic method and constrained optimization methods is discussed in [5, 6]. Other computational intelligence methods and classical algorithms for energy management of microgrids are discussed in [7].

All of these researches address microgrid operation problems in a centralized manner. Traditional Supervisory Control And Data Acquisition (SCADA) system has a central controller, which gathers all system knowledge and makes the decision. Dynamic adaptation leads to complication in centralized systems. Moreover this central coordinator will provoke considerations about scalability, computational complexity, and communication overhead. But the MAS architecture can reduce these concerns by following decentralized approach. A multiagent solution to energy management in hybrid renewable energy generation system is discussed in [8]. Agent based control frameworks are discussed in [9]. An agent based operation of microgrid is discussed in detail in [10]. Here the capabilities of MAS to improve the operational efficiency are analyzed. Optimization of microgrid with intermittent renewable energy resources using MAS is discussed in [11]. Here, MAS approach is used in a dynamic environment to distribute decisions related to microgrid operations and eventually to improve performance and stability. But decentralized approach of MAS is not exploited here. MAS technology is used for controlling microgrid operations and also for optimal energy exchange with the grid as discussed in [12]. Fully decentralized approach of MAS for microgrid operation and participation in energy market is discussed here. But each agent should have complete knowledge about the environment and decision making capabilities to be fully autonomous. It is not economical to be fully autonomous. Also we want to use agent communication for coordinated action. So some level of centralized control is necessary for coordinated operation. So a hierarchical approach of MAS for microgrid operation is considered in [13]. Here, the design and implementation details of MAS in microgrid energy management and MAS facilitating the seamless transition from grid connected to an island mode are also discussed. But linking MAS with MATLAB is not explained in detail. Real-Time Digital Simulator (RTDS) is used for real-time operation of MAS on microgrid is discussed in [14]. Here MAS system was implemented in JADE platform and was interfaced with RTDS via TCP/IP protocol. But sensing all the dynamic variations and the control strategies are not addressed here.

Multiagent based distributed energy management for intelligent microgrid is discussed in [15]. Here MAS simulated for market operations of integrated microgrid is discussed in detail. Practical realization of MAS for market operation is not attempted in this paper. A comprehensive power management in microgrid with distributed agents is discussed with case studies in [16]. The various trends in microgrid control are discussed in [17]. The various control strategies to improve stability of microgrid are discussed here. The complete review of microgrids in MAS perspectives is discussed in [18].

Distributed online optimal energy management for smart grid is discussed in [19]. Here MAS is used for interactive operation of economic dispatch and demand response for maximizing the social welfare. MAS simulations implemented through JADE are discussed here. Multiagent system implementation for home energy management is clearly explained with case studies in [20]. But the hardware implementation details are not given. A detailed review on agent concepts applied to intelligent energy systems is given in [21].

Real-time microgrid control strategies are discussed in very recent paper [22]. The control strategies to optimize the power exchange between microgrid and main grid are discussed here. But facilitating MAS for real-time operations of microgrid is not discussed in detail. In most of the references, MAS simulations of microgrid operations are implemented in JADE platform and the real-time operation of MAS is not adequately addressed. In order to practically realize the MAS operations in the field, MAS has to be linked with MATLAB/Simulink model to sense the environment variables and the outcome of the multiagent operations should be given back to the actuators through MATLAB/Simulink for physical action. But MAS operations in JADE cannot be linked with MATLAB, as MATLAB does not support parallel operations, which is essential for a decentralized approach such as MAS. So we consider a novel approach, Multiagent Control Using Simulink with Jade Extension (MACSimJX), to link MATLAB with MAS to allow system designed with Simulink to be controlled by agents that operate in JADE, a powerful development environment for modeling MAS [23]. A decentralized multiagent system approach for service restoration using islanding of distributed generator is discussed in [24]. Only outage management is discussed in detail here. The other challenges of integration of renewable energy resources and the controllable loads are not considered. Latest survey on multiagent systems for microgrid control is given in [25]. The papers considered in the survey explain the idea of practical implementation of MAS but a hardware ready model is not clearly addressed in any of the papers. Very recently the integration of JADE with Simulink using MACSimJX is discussed for energy management in distributed energy resources (DER) of microgrid in [26]. However, the agent programming concepts, linking MAS with MATLAB/Simulink model and strategies for sensing and controlling the dynamic variations of the environment, are not adequately addressed in this paper.

The shortcomings of the related works which bring the motivations for the proposed approach are given below.

- Previous research related to microgrid energy management using MAS has mainly focused on prototype design and simulation of distributed control methods but the need to test and validate the implemented applications, in a coded form ready for deployment, has not been adequately addressed.
- MAS linking MATLAB for microgrid energy management is not discussed in detail. Time synchronization in cosimulation between MAS and MATLAB is not explained in detail.
- All the options available for effective energy management of microgrid has not been considered.
- The impact of stochastic nature of the renewable resources on the microgrid operations and the mitigation process are not adequately addressed. Also problems of stability and reliability due to increased penetration of renewable energy resources are not considered.
- Microgrid is building block of smart grid and so all the features of smart grid can be implemented in microgrid. But only energy management is discussed in most of the works. Controllable loads, dynamic pricing, and plug and play features are not discussed so far in the microgrid environment.
- The process of development, verification, and deployment of time-critical MAS applications has not been adequately addressed to date.
- Multiagent system features such as runtime adaptive behavior, less communication overhead, and less computational demands are not adequately exploited for microgrid energy management applications.

In the proposed approach, all the above-mentioned shortcomings are addressed adequately. Here a comprehensive approach is used, considering all the options available in a microgrid for improving the energy management efficiency in a dynamic, distributed environment. In this approach, MAS is implemented in JADE and then it is linked with MATLAB/Simulink, using Multiagent Control Using Simulink with Jade Extension (MACSimJX). This opens MATLAB to one of the most strongly supported open source agent development environments, allowing real-time hardware systems to be modeled alongside and interacting with software model such as multiagent systems, bringing MAS closer to practical implementation. Here, the solar microgrid system designed with Simulink is controlled by MAS agents that operate in JADE. MAS in JADE is fully exploited for control and automation in distributed, dynamic energy management of solar microgrid, considering the intermittent nature of solar power, randomness of load, and dynamic pricing of grid. New control strategies are formulated through generating System State Number (SSN) and Control State Number (CSN) to sense all the environment variable values dynamically every hour and reflect these values in JADE agents for taking strategic action to maintain the stability and improve reliability of the microgrid with stochastic nature of solar power and load. Also demand side management strategies are implemented autonomously by controlling of noncritical Load (NCL). The automated application is represented as an agent control application where each microgrid component is represented by an agent and the task of the agent is to process the environmental information, received from Simulink, and optimize the microgrid operation through autonomous actions and also collaborating with other agents for strategic decision.

The rest of the paper is organized as follows. Solar microgrid is explained in Section 2. A detailed discussion on multiagent system approach and multiagent platform is given in Section 3. Implementation of dynamic energy management of solar microgrid in distributed environment using JADE is given in Section 4.  Section 5 deals with agent formulations and Section 6 deals with implementation of control operations of solar microgrid for demand side management, using MACSimJX. Case study, simulation, and validation are given in Section 7. Conclusion is given in Section 8.

## 2. Solar Microgrid

**Figure 1:**
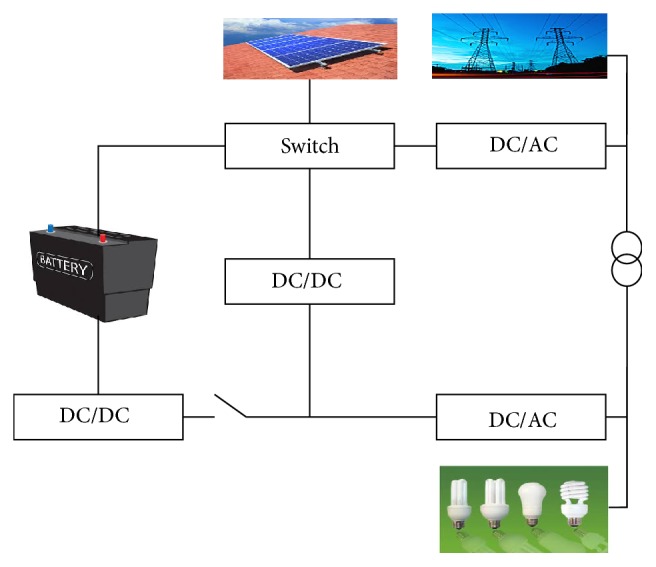
Solar microgrid.

The architecture of the considered solar microgrid is shown in Figure 1. The consumer can cover his demand partly by using the electricity produced by the local renewable (solar) generator, partly by stored electricity in the battery when the solar source is not available. The battery is charged when the solar source is available, and can discharge the storage when needed. The consumer has the possibility to control the storage and the solar power generator. The challenge in solar microgrid is that the power supply is intermittent in nature and with the ultracapacitors and battery the randomness in the supply should be managed so that the load is given constant supply all the time irrespective of the nature of the solar power. The battery can be charged by the solar power or from the grid.

A solar PV module consists of a number of solar cells connected in series or parallel based on the requirement. There are various factors which affect the solar power like solar irradiance, temperature, partial shading and cloud, arrangement of the cells, and the angle of tilt of the panel. The maximum power is found by Maximum Power Point Tracking (MPPT) algorithm [27]. The MPPT refers to the point with a maximum power output in the curve under specific external temperature and solar irradiation.

## 3. Multiagent Systems Approach

### 3.1. Multiagent Systems

Autonomous components and coordination are the basic ingredients of any distributed system. Distributed systems that involve many heterogeneous entities have some major limitations:

- Interactions among participating entities are fixed by application developer while coding and hence they lack runtime adaptive behavior.
- Some applications have to operate in environments where maintaining continuous communication is expensive. So distributed systems with many ongoing interactions are almost infeasible.

These considerations have motivated the development of approaches to distributed system based on agents which provide ways for adaptation and ongoing interaction. A multiagent system (MAS) is a distributed system consisting of multiple software agents which form “a loosely coupled network,” to work together to solve problems that are beyond their individual capabilities or knowledge of each entity.

Multiagents overlay a way to elaborate systems that are decentralized rather than centralized, emergent rather than planned, and concurrent rather than sequential. MAS has inherent benefits such as flexibility, scalability, autonomy, and reduction in problem complexity. Agents have certain behavior and tend to satisfy certain objectives using their resources, skills, and services. Ability to produce is a skill and selling power to grid is a service. The way that the agent uses its resources, skills, and services defines its behavior and the behavior of each agent is formed by its goals. The MAS approach's goal is to control a very complicated system with minimum data exchange and minimum computational demands.

**Figure 2:**
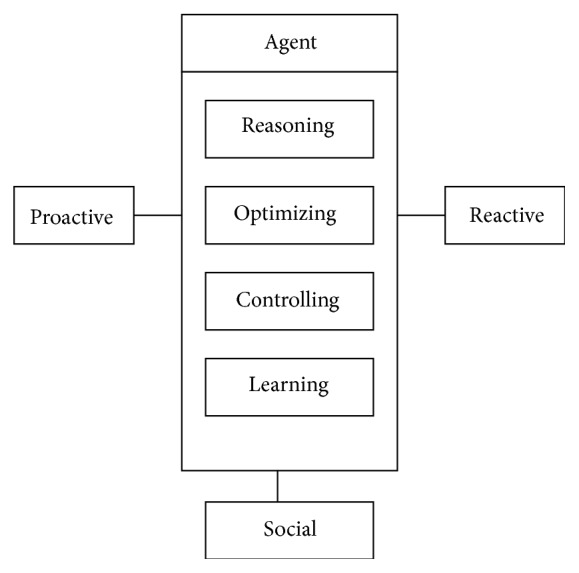
Functionality of an agent.

In MAS, several autonomous and intelligent entities called agents are working in collaboration to achieve the overall goal of a system. Agents have four behavioral attributes, autonomy, social, proactive, and reactive. Autonomy refers to the principle that agents can operate on their own to meet their goals without the need for human guidance. Agents are proactive, that is, the ability to take the initiative rather than acting simply in response to their environment. Agent can cooperate with other agents for coordinated action. In order to cooperate, agents need to possess social ability, that is, the ability to interact with other agents with some communication language like Agent Communication Language (ACL). Agents are reactive to changes in environment. Reasoning, optimizing, controlling and learning are the inherent characteristics of an agent, as shown in Figure 2. For agent systems to be truly smart, they would have to learn as they react and/or interact with their external environment.

### 3.2. Multiagent Systems in Microgrid

Centralized SCADA, which was originally designed for traditional passive networks, may be inadequate to cope with the high penetration of DERs and complex control decisions due to its centralized approach and lack of flexibility and extensibility. In the microgrid, uncertainty in SCADA systems arises when sensor data or inferred knowledge cannot be deemed accurate due to intermittent nature of DERs. Also SCADA has problem dealing with inherent noise/error in sensor data or knowledge as well as uncertainty, incompleteness, and inconsistent or conflicting data from multiple, heterogeneous sources. Humans have traditionally supervised such problems to reason and resolve issues. A multiagent Energy Management System (EMS) can cope with heterogeneity and give better, faster solution than SCADA, taking the automation of microgrid to the next level. With decentralized approach, a MAS has its own perception of the environment, goal, and agenda and it tries to achieve the best for itself while behaving strategically.

MAS can deal with disadvantages of SCADA and increase the operational efficiency of solar microgrid due to its inherent characteristics and functionalities taking the automation of microgrid to the next level. MAS are by nature distributed and concurrent; they are independent entities engaged in the system; microgrid is tightly associated with the communications between stakeholders and entities (agents) to exchange information. Plug and play adaptability and connection to external grid are seamless in MAS based microgrid. By nature, MAS can be scaled up by adding other agents or by dispersing them in new environment with new resources and capacities. MAS is particularly useful for designing distributed systems requiring autonomy of their entities.

The development of smart grid and related technologies combines advances in distributed systems, artificial intelligence, control, and information and communications technologies. MAS in mirogrid leads to high level of autonomy, self-healing, and reliability and leads to providing features such as reconfiguration, protection, restoration, and interaction with other users through demand response.

In MAS, set of economic and control mechanisms is used for dynamic balance of supply and demand across the entire electrical infrastructure using value as a key operational parameter.

### 3.3. Multiagent Platform

**Figure 3:**
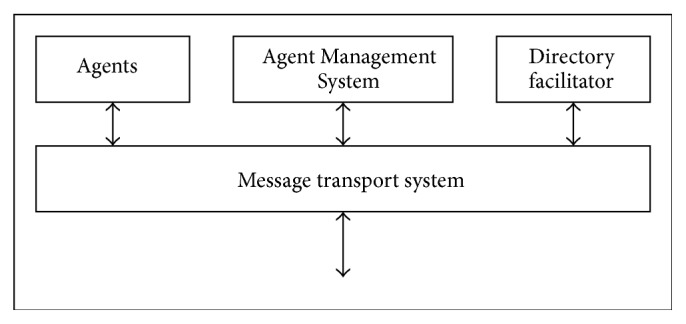
Architecture of multiagent system.

JADE provides a convenient distributed platform for users to focus on developing agents for control and monitoring of power balance during microgrid operation. JADE is an open system which supports plug and play capabilities and is also scalable without much modification to the control scheme. In this paper, JADE framework that conforms to FIPA (Foundation of Intelligent and Physical Agent) standard for intelligent agents is used. Agent platform is a software environment, where software agents run. Agent lives in a container, and a collection of containers make up a platform. Agent Management Service (AMS) is responsible for managing the agent platform, which maintains a directory of Agent Identifiers (AIDs) and agent states. Each agent must register with an AMS in order to get a valid Agent ID. Directory Facilitator (DF) provides the default yellow page services in the platform which allows the agents to discover the other agents in the network based on the services they wish to offer or to obtain. A MAS architecture is shown in Figure 3.

JADE is also used as the runtime environment in which agents execute, thereby masking from the agents the underlying complexity of the operating system or network. Agents can span multiple computers or be on one computer, yet for the implementation, the code for sending and receiving messages is the same. A platform encompasses all the containers within an agent system. JADE contains main containers and peripheral containers. When a platform is created, the main container is always the first container to be initialized in JADE. Agent Management System (AMS) and Directory Facilitator (DF) agents are also automatically created once the main container is initialized. The directory services and other administration services are hosted on the main container, which is the first container launched in the platform, but are duplicated on the other containers for robustness. JADE platform provides a set of functions and classes to implement agent functionality, such as agent management service, Directory Facilitator, and message passing services [28].

## 4. Implementation of Multiagent System Based Energy Management of Solar Microgrid with JADE

**Figure 4:**
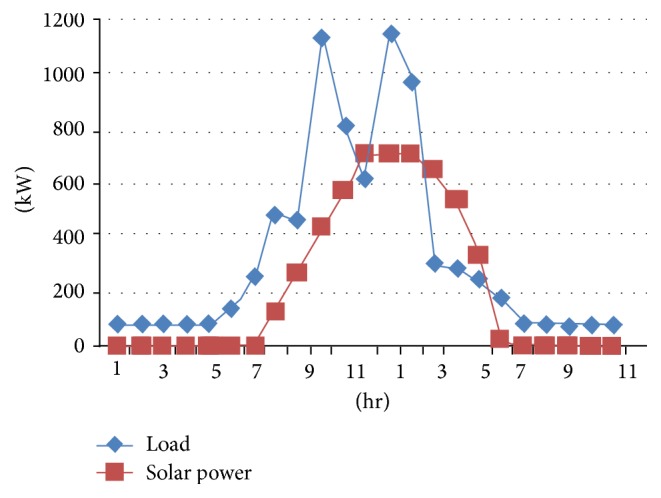
Solar power and load for department.

**Figure 5:**
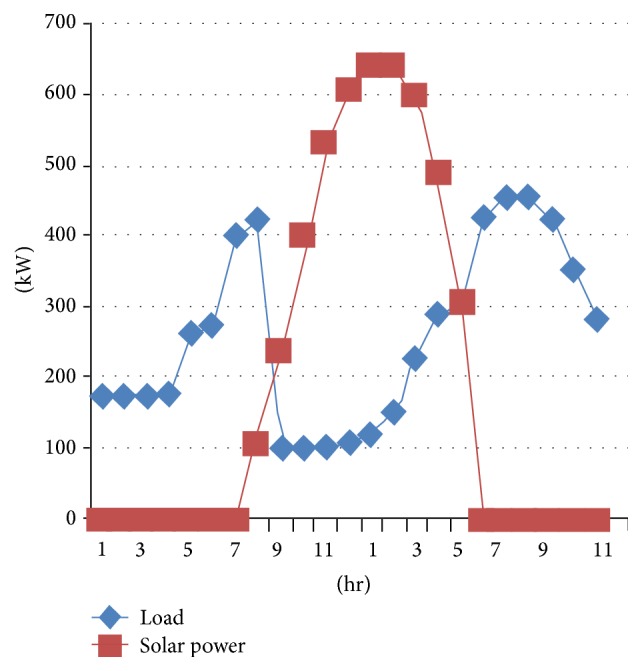
Solar power and load for hostel.

The proposed multiagent system is implemented using the Java Agent Development Environment (JADE). JADE is a Java-based open source software framework for developing multiagent systems. The JADE architecture is built on peer-to-peer modality. Intelligence, initiative, information, resources, and control can be fully distributed across a group of heterogeneous hosts and devices, through wireless or wired networks. Each agent can communicate and negotiate with its peers to reach mutually acceptable agreements for cooperative problem solving. We consider a grid connected solar microgrid system containing two solar photo voltaic (PV) systems, one in the department of the college and the other in a hostel with the capacity 1000 kW each, each of which contains a local consumer, a solar PV system, and a battery. Every hour solar power is taken from NREL (National Renewable Energy Laboratory). All the electrical appliances are considered for per hour load calculation based on their usage for the department and hostel. The solar power and the load graphs of department and hostel are shown in Figures 4 and 5.

**Figure 6:**
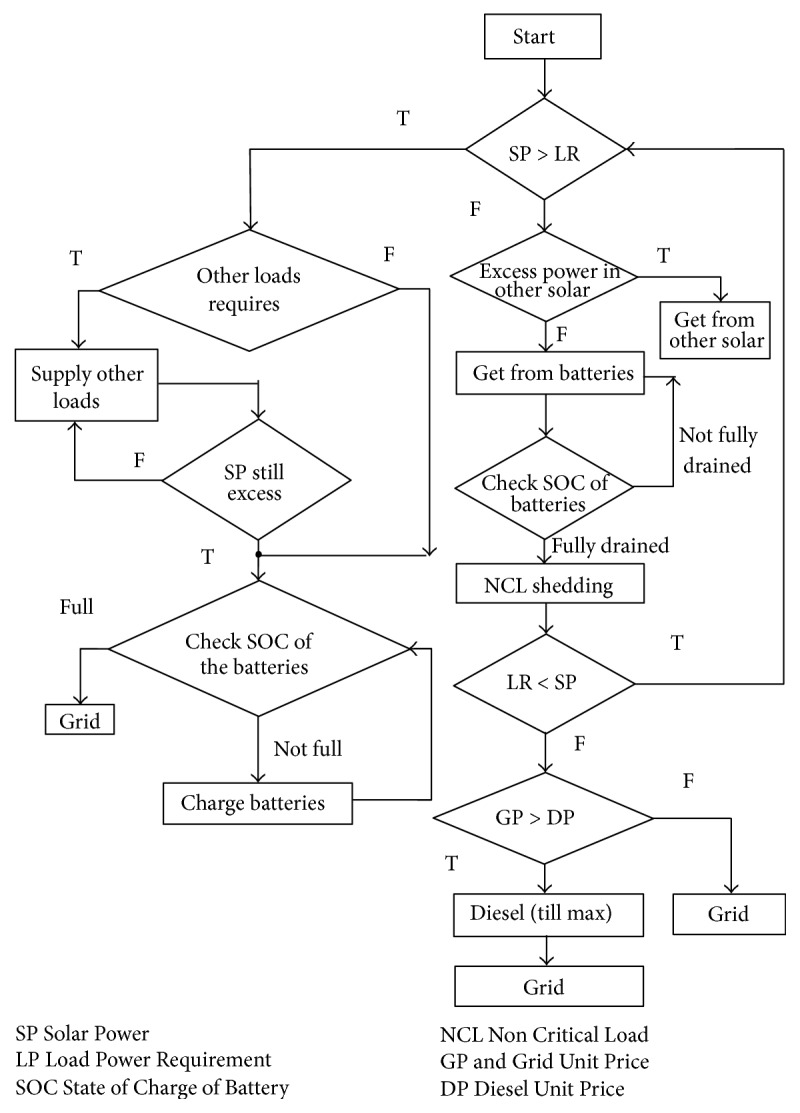
Flowchart.

The solar power, load, state of charge (SOC) of the battery, noncritical loads, and dynamic pricing of grid are monitored hourly, and based on these data, the agent takes best possible actions autonomously for dynamic energy management of the solar microgrid in a distributed environment. Considering all the possible options available for the solar microgrid, a flow chart is drawn as shown in Figure 6. The proposed system has the following agents: solar power generator agent, load agent, grid agent, diesel agent, and control agent. Each PV system has all these agents. Multiagent programming is done in JADE in Eclipse environment. The overall procedure is the following.

1. Here first the department load agent (LDA) communicates the power demand through an ACL message with the available solar power in department solar agent (SDA) in the department at that specific hour. If surplus power is available in SDA, SDA takes autonomous decision to give power to LDA. The remaining power is used to charge BDA and BHA through control agent. After charging the batteries, the excess power is given to the grid through control agent. Thus, the load agent makes the local decision as well as communicates with other agents for global decision.
2. If power available in SDA is not sufficient for covering load, it checks with the availability of solar power in the hostel solar agent (SHA). If required power is not available then it looks into the battery of the department (BDA). If required power is fully available it takes from BDA and if partially available, SDA checks with the BHA. If required power is available, it is taken.
3. If the power is still required after taking from solar unit and battery, SDA checks for noncritical load shedding through control agent for implementing demand side management strategies. Load response strategies include both load shedding and load shifting. Load shedding involves curtailing equipment that is not mission critical and load shifting is the rescheduling of energy-intensive operations to a different time period. Noncritical loads can have many priorities based on the requirement. Even after this, if load requires power, it check with the pricing of the grid at that hour and the diesel power price and chooses the least priced one.
4. Every hour based on the load requirement and availability of solar power the agent makes the best possible decision for economic operations in a distributed environment.
5. Similar steps are followed for the load agent in the hostel. All the communication is done through ACL. Thus, every hour the solar microgrid energy management is done dynamically for distributed optimization of solar microgrid by using multiagent system in JADE platform. Programming is done for every agent in Java in JADE platform and executed in Eclipse environment. All the communication between the agents is done through Agent Communication Language (ACL). The complete interactions are shown in the sniffer diagrams. The console output gives the transaction report of a particular scenario.

## 5. Agents Formulations

The proposed multiagent system comprises many intelligent agents representing various components in a microgrid. Each agent has a localized knowledge base, containing rules and behaviors, which governs its decision making process.

The following agents are formed to simulate multiagent system in JADE environment.

### 5.1. Generator Agent

Generation agent (GA) receives power request from load agent. It allows owners of solar generators to set selling price for trading. The initialization for all agents begins with registering itself with the Directory Facilitator (DF) by providing it with a set of service descriptions like to setName and setType. For GA, setName and setType are chosen to be solar power source and power selling, respectively. Department solar agent (SDA) and hostel solar agent (SHA) are considered in our works.

### 5.2. Load Agent

Load agent allows customer to specify the amount of power to purchase and communicate with the generator agent. The load agent is taken as a buyer agent because it searches the DF yellow pages for generation agents offering power supply services. It first registers itself with DF by providing load and power buyer as input parameters to setName and setType, respectively. If there are no GA in the DF yellow pages, it checks for batteries and finally ends with control agent (CA) buying power from the grid to meet its load demand. Thus, load agent makes local decisions and communicates and collaborate with other agents for collective decision. Department load agent (LDA) and hostel load agent (LHA) are considered in our case.

### 5.3. Grid Agent

Grid agent (GA) registers with DF by giving grid agent and grid status as inputs to setName and setType, respectively. Grid agent collects real-time grid pricing and informs other agents about connection status of grid. Grid price varies every hour leading to dynamic pricing concept of smart grid. The main set of behaviors for grid agent is receiving request from CA. This behavior will wait for messages from CA to determine whether the microgrid needs to buy power or sell net surplus power to the grid during grid connected mode. Then, it gives the power or accepts the power according to control agent instructions.

### 5.4. Diesel Agent

Diesel agent registers with DF by giving diesel agent and diesel status as input to setName and setType, respectively. It gives the price of per unit power. It receives request from the CA and sells power to load agent when the diesel price is less than the grid price.

### 5.5. Control Agent

Control agent (CA) is responsible for monitoring, controlling, and negotiating power levels and performing power exchange between the solar microgrid and main grid. Initially CA registers as control and microgrid control management as inputs to setName and setType, respectively. The control agent (CA) displays the total microgrid power generation and loading as well as computing the net microgrid power every hour. Then, the agent will determine whether to buy or sell power to the grid based on the value of net power. If the grid power unit price is greater than diesel generator (DG) unit price then it prefers to buy from DG.

### 5.6. Agent System and the Agent's Relationship

Agents sense the environment and make simple decisions which are within their capacity. For complex decision, agents communicate with control agent and inform the requirements. For example, department solar agent gives power to the department load requirement if it is having enough power. If there is a shortage of power, it informs the control agent about the power requirements. The control agent gets the status of all the agents from Directory Facilitator (DF). where all the agents are registered with their services. It validates the request made by other agents and informs other agents about the best place to get the requirement for optimal energy management in the solar microgrid.

**Figure 7:**
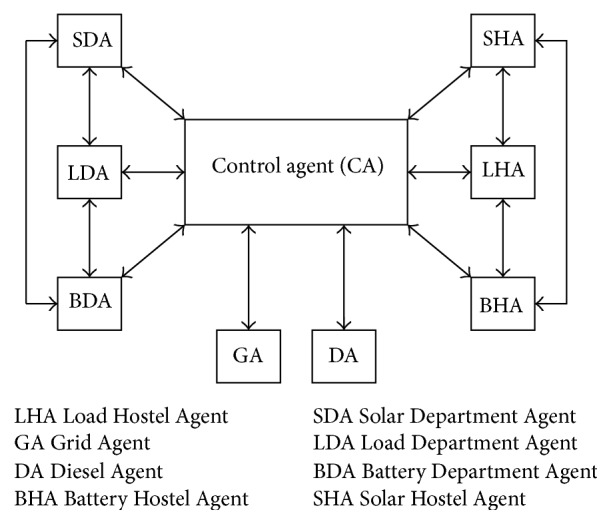
Agents relationship diagram.

The agents relationship diagram is shown in Figure 7. Here, the load agents participate in the system as buyers of energy, while the solar generator agent participate as sellers of energy. Here the hierarchical approach, which combines centralized and decentralized approach, is used. Every agent autonomously makes decision then through control agent it communicates to other agents for strategic decision. We consider solar department agent (SDA), load department agent (LDA), battery department agent (BDA), solar hostel agent (SHA), load hostel agent (LHA), battery hostel agent (BHA), grid agent (GA), diesel agent (DA), and control agent (CA). Every hour based on the net power availability and the load requirement, the transaction with the grid is made by the CA. When LDA request power from SDA, SDA gives power to LDA autonomously. If surplus power is available, it is given to BDA. Further excess power is given to BHA and finally to the grid agent (GA). The validation is done through control agent. If there is no enough power available in SDA then LDA contacts SHA and receives the available power. If the power is still required it contacts the BDA and then BHA and finally it communicates with control agent to do the NCL shedding and finally the post-NCL shedding power is received from grid or diesel agent based on the unit price at that point of time.

Similarly, the LHA contacts SHA for power. SHA makes the autonomous decision to give the power to LHA. If surplus power is available after supplying to LHA, SHA checks the BHA and BDA for charging and further excess power is given to grid. If SHA does not have enough power required by LHA, LHA contacts SDA and gets the power available there. If power is required still, LHA contacts BHA and BDA to get the power available in the battery. The further power required is managed by contacting the control agent which does the NCL shedding and finally the post NCL shedding power is received from grid or diesel agent after comparing the unit prices.

### 5.7. Simulation Results and Case Study

One round of operation of solar microgrid was simulated for 24 hours, considering all the possible scenarios happen in a day. All the operations are considered as shown in the flow chart and for these scenarios, the Java programming is done for all the eight agents of the solar microgrid in JADE framework and executed in Eclipse Integrated Development Environment. Various scenarios are considered and sniffer diagrams and the console output representing the interaction of the agents and transaction details are studied.

The solar power and the load for the department and hostel at 8 am are considered for the case study. The solar power of department and hostel is 200 kW each and the load in the department and hostel is 500 kW and 200 kW, respectively. The sequence of operations is given below.

1. Department Load needs 500 kW but taps 200 kW from the available department solar power as department solar power available is only 200 kW. Still it requires 300 kW.
2. Department load checks with hostel solar power. But in hostel no excess power is available as the load requirement is equal to the available solar power of 200 kW. Department load then checks with the battery of department and hostel but no power is available in the battery.
3. Department load then goes for noncritical load shedding before going to grid or diesel for demand side management. Noncritical load in that particular hour is 200 kW. So it sheds 200 kW and still it requires 100 kW.
4. Finally, it checks with the unit price of grid and diesel. In the dynamic pricing of grid the unit price that hour is Indian Rupees 8/kWh and the diesel unit price is Indian Rupees 10/kWh. It chooses the least priced one, which is grid, and so it gets the remaining power of 100 kW from grid.

The console outputs and sinffer agent diagram are shown in the following.

*Output*

*Department*

 Power: 200 kW
 Load: 500 kW
 Power tapped from local agent: 200 kW
 Power tapped from the other agent: 0 kW
 Power tapped by battery: 0 kW
 Battery charge: 0.0%
 Power remaining: 0 kW
 Power needed: 300 kW
 Noncritical load shed: 200 kW
 Power needed after load shedding: 100 kW
 Preferred nonrenewable power source: grid
 Power tapped from grid: 100 kW grid price/kWh = Rs.8
 Total power tapped from grid: 100 kW

*Hostel*

 Power: 100 kW
 Load: 500 kW
 Power tapped from local agent: 100 kW
 Power tapped from other agent: 0 kW
 Power tapped by battery: 0 kW
 Battery charge: 0.0%
 Power remaining: 0 kW
 Power needed: 400 kW
 Noncritical load shed: 100 kW
 Power needed after load shedding: 300 kW
 Preferred nonrenewable power source: grid
 Power tapped from grid: 300 kW grid price/kWh = Rs.8
 Hostel load tapped 100 kW from hostel solar
 Department load tapped 200 kW from department solar
 Hostel load tapped 0 kW from department solar
 Department load tapped 0 kW from hostel solar
 Hostel load tapped 0 kW from hostel battery
 Department load tapped 0 kW from department battery
 Hostel load tapped 0 kW from department battery
 Department load tapped 0 kW from hostel battery
 Hostel load tapped 300 kW from grid
 Department load tapped 100 kW from grid

After trading and negotiation are completed, the final result is being reported by control agent, which tells users how much power is being traded with the grid.

## 6. Practical Realization of MAS in JADE for Dynamic Energy Management of Solar Microgrid Using MACSimJX

### 6.1. Multiagent Simulation Using MACSimJX

**Figure 8:**
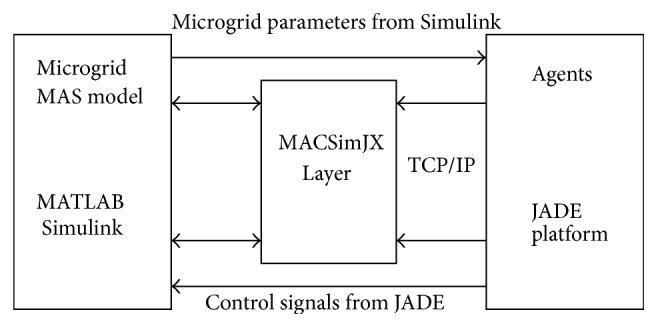
Multiagent system with MATLAB.

A microgrid using a MATLAB/Simulink structure can effectively achieve energy management and demand side management through JADE, allowing each component in the microgrid to communicate and obtain the status of the entire network at any time. The component status of MATLAB/Simulink can be sent to the JADE agent system. If the breaker needs to switch after judgment, the JADE agent system will send a signal to the corresponding breaker in MATLAB/Simulink. When unexpected situations occur in the microgrid, JADE can immediately respond and manage the situation; for example, it can rapidly disconnect the power source in island mode and integrate distributed energy into the grid to provide power supply. Once power has been restored to the source, it can be reconnected with the system immediately to provide power. The use of multiagent systems in microgrids is extremely flexible because the monitoring and management can be adjusted to microgrids of various structures and demands. The Simulink model is developed for the solar microgrid with solar power, load, and battery. In Simulink, the S-functions are unable to handle multiple threads of execution, which is an essential characteristic of MAS: they become unstable if several processes run concurrently inside Simulink. To overcome this problem, MACSimJX, which acts a middleware between Simulink models and the agents, is used to bring MAS closer to the physical models as shown in Figure 8. MACSimJX has a client-server architecture, separating the MAS from Simulink. Client is in the Simulink and the server is at the agent environment.

Named pipes in windows are used for communication [29]. Two pipes are used, one for passing configuration information and the other for passing simulation information. The MAS simulation framework has the following characteristics:

- A fixed-rate, discrete time, simulation with no explicit internal state.
- True multithreading in parallel with Simulink's simulation cycle.
- The capability to synchronize with Simulink operations.

**Figure 9:**
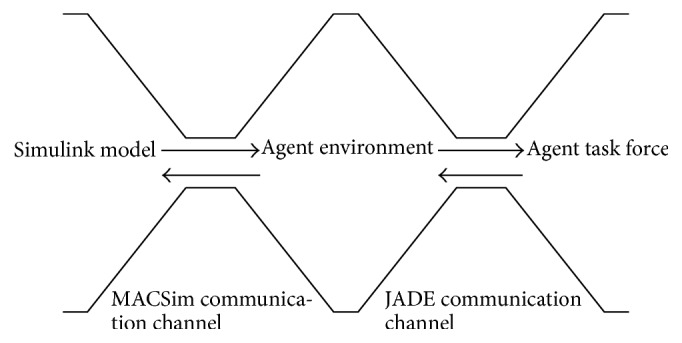
A complete model of MACSimJX.

Initially the environment variables of solar microgrid are sensed by the sensors installed in all the physical devices of the solar microgrid. The complete model of MACSimJX is shown in Figure 9, which shows how the signals are given from MACSim client to the MACSim server. Once simulation signals arrive at the MACSim server, they are passed on to the agent model, which is divided into two parts, agent environment and agent task force (ATF). The agent environment provides essential facilities such as coordination and messaging. The agent environment has an agent coordinator and an agent server. The agent coordinator posts messages to ATF which assigns the work to various agents, requesting them to carry out any operations necessary to prepare outputs for the specified time step. The ATF consists of all the agents that jointly operate on the data arriving from Simulink in order to accomplish the goal of optimizing the energy management of solar microgrid. The decentralized approach of MAS with the runtime efficiency of JADE improves the performance and reduces the computational complexity. Then, the computed outputs are passed back to the agent environment. Once the agent environment has the completion messages from all agents, the output values are passed to the pipe server to be returned to Simulink. The messaging, therefore, provides synchronization with Simulink's simulation cycle so that the messages can be sent as command signals to the actuators for switching operation of microgrid devices.

**Figure 10:**
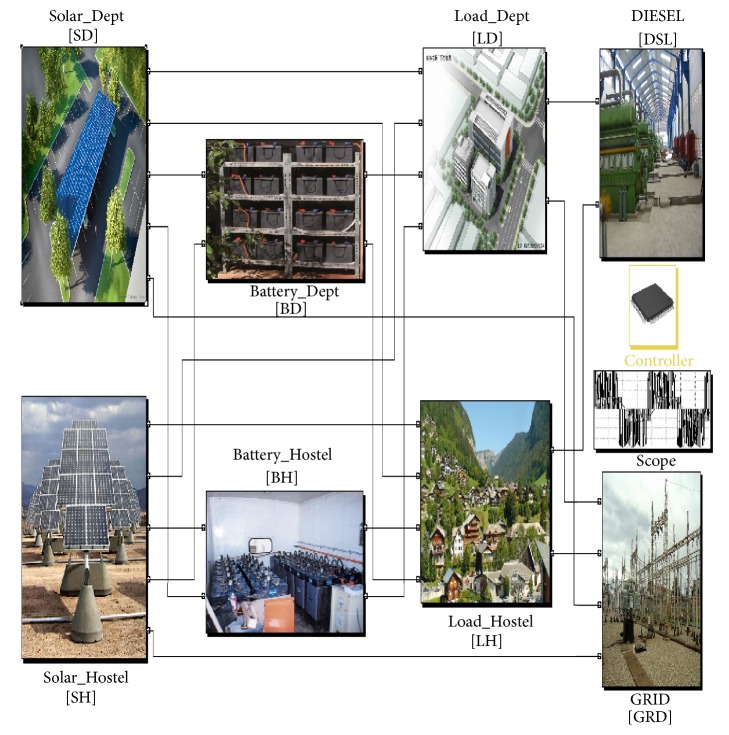
Simulink model of solar microgrid.

This paper aims at dynamic energy management by distributed automation of solar microgrid in a distributed environment. The important components of solar microgrid are programmed in agents so the component status of MATLAB/Simulink can be sent to the JADE agent system. If the breaker needs to switch after judgment, received after operation of agents, the JADE agent system will send a signal to the corresponding breaker in MATLAB/Simulink, which in turn is given to the physical breaker for real-time action. The approach considers a two-layer framework comprising agent layer containing the agent based control and functional layer, where the physical infrastructure is simulated in MATLAB/Simulink. The behavior parts of the agents (i.e., decisions and control strategies) and coordinating functionalities are done in the agent layer aiming to optimize microgrid operation. The functional layer includes the model of the real solar microgrid comprising solar units load, batteries, diesel unit, and grid. MACSimJX acts as a middleware linking these two. The functional layer gets the values of the environmental variables and passes them to the agent layer. The agent layer observes values and coordinates with required agents using the decentralized approach of MAS operating in JADE and takes necessary actions for optimizing the solar microgrid under dynamic environment. The agent layer needs to interact with the function layer to have a mean to test the control strategies. The agent layer consists of department load (LDA) and solar (SDA) and battery agents (BDA) and hostel load (LHA) and solar (SHA) and battery agents (BHA) apart from grid (GA) and diesel agents (DA). The control agent (CA) monitors and controls all the agents. The agent layer uses MATLAB/Simulink as gateways to translate semantics from agent world to services world, where the commands can be physically executed. The quality of service and the dynamic energy management are done by proper control and management strategies, which accommodate different heterogeneous entities and also remain secure, sustainable, and reliable. The MATLAB/Simulink model of the solar microgrid is shown in Figure 10.

### 6.2. Simulink Model of Solar Microgrid

The Simulink model is developed for SD, LD, BD, SH, LH, BH, GRD, and DSL individually. In the battery model, three types of state of charge (SOC) are considered. SOC is full, SOC is between full (100%) and cut-off value (40%), and SOC is below cut-off value. The lesser the available power, the longer it takes for the battery to fully charge, and vice versa. Similarly, if more power is drawn, it discharges in short time and if less power is drawn it discharges for long time. Like this model for solar power, load, grid, and diesel are developed. The abbreviations used are shown in Table 1. The input port and output ports are defined in simulation model of solar microgrid. Eight input ports and 18 output ports are considered. These 18 output ports are connected to the actuators for physical action.

All the components are considered for optimal energy management, such as solar power, load power, and the state of charge of battery. Based on all possible environmental parameter values of solar microgrid components, a unique number is formed, which is broadcasted to all the agents. By observing it, each agent decides the subset of switching operations it is expected to do since it is already supplied with the consolidated state numbers while programming. It observed the state number and if it is present in its consolidated list of state numbers it will do the switching operations of the corresponding switches; else, it will not respond.

## 7. Case Study

### 7.1. Case Study with Critical Load

The Simulink modeling is implemented for solar power unit, load, and batteries of the department and hostel and grid and diesel power. Here BD and BH refer to department and hostel battery SOC. SDP, LDP, SHP, and LHP refer to solar power and load values of the department and hostel. DPR and GPR refer to the diesel price and grid price in INRS (Indian Rupees). The input values taken for the case study are shown in Table 2. All the agents programs are executed in JADE and then MACSimJX is executed to link the agent environment with Simulink model. Then, the solar microgrid Simulink model is made to run. The environment values are given to the JADE agents through the Simulink and after the coordinated operations of agents, the results are given as command signals to the Simulink for the switching operations. The agent senses the environment through Simulink and takes the strategic action for optimal energy management of the microgrid in the dynamic environment. Every hour the environment is changing due to intermittent nature of solar power and the variation of load. The agent takes the best possible action every hour.

**Figure 11:**
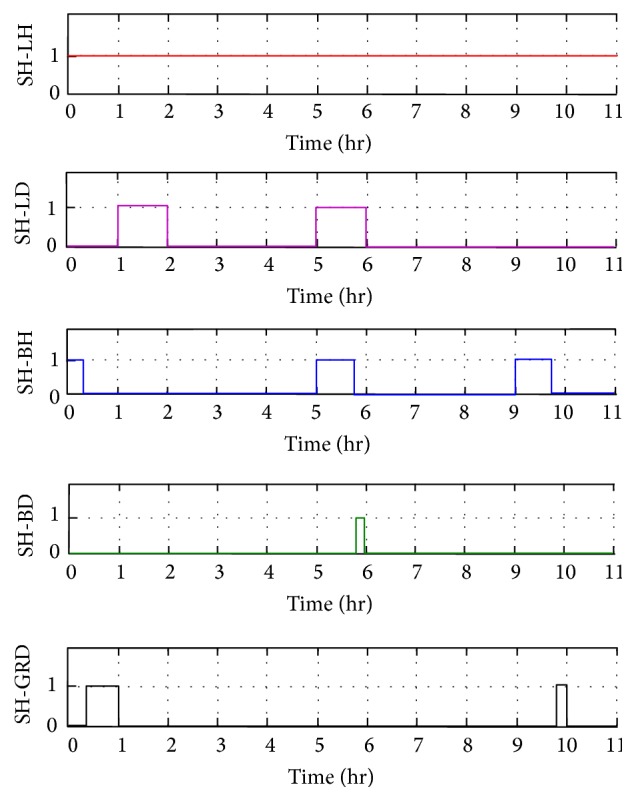
Switching operations of solar hostel agent (SHA).

**Figure 12:**
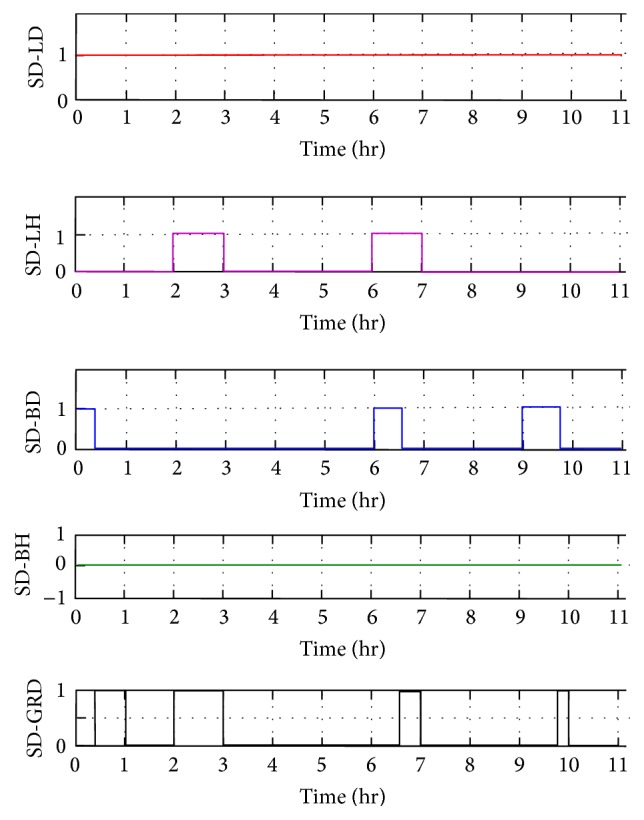
Switching operations of solar department agent (SDA).

Every hour based on the solar power and load values the optimal distributed energy management is implemented by the strategic action of agents. By using multiagent system implemented in JADE, the runtime efficiency of the solar microgrid is improved. Thus, dynamic energy management and demand side management are done for all the possible scenarios in this case study. The individual switching action of all the agents is analyzed. For example, the switching action of the solar hostel agent (SHA) in critical load case study is shown in Figure 11. Here the switch SH-LH is always on as the local load LHP is always supplied by SHP. Initially from 0th to 1st hour, the department and hostel solar powers (SDP and SHP) are 200 kW more than the corresponding load demand and so the department and hostel batteries are charged. The solar hostel agent charges its battery BH by switching on SH-BH and after BH gets fully charged the power is given to grid by switching SH-GRD. From 5th to 6th hours, department load requires 300 kW but the available solar power is 200 kW. So the 100 kW deficiency in the department is managed by 200 kW excess in the hostel unit. The hostel unit gives 100 kW to the department load and the remaining 100 kW is used to charge the hostel battery first and then the department battery. Here SHA switches on its local load (LHP) and also switches on SH-LD to give 100 kW deficiency in the department load. The remaining 100 kW is used to charge its battery (BH) by switching on SH-BH. After hostel battery gets fully charged, the switch SH-BH is switched off and SH-BD is switched on to charge the department battery (BD). Similarly, the switching actions of all the agents are observed. The switching operation of the department solar agent is shown in Figure 12. Here SD-LD switch is always on as department load is always connected to the department solar power. From 2nd to 3rd hours, there is deficiency of 100 kW in the hostel solar in supplying to hostel load. So it receives 100 kW from department solar through SD-LH switch. In the 6th hour, it is again switched on to manage the deficiency in hostel solar unit. In the first hour, the excess power in the department solar is used to charge department battery by switching on SD-BD till it gets fully charged and then the power is given to grid by switching on SD-GRD. It is again switched on from 6th hour till it gets fully charged and then the power is given to grid by switching on SD-GRD. In the 9th hour, it is switched on again to charge as there is excess power in the department solar unit and then it is given to grid through SD-GRD. Simulation output of all the components of the solar microgrid is observed and the actions are verified. The Simulink model output can be given for real-time physical action through Intelligent Electronic Devices (IED) and Program Logic Controllers (PLC). Thus, microgrid quickly adapt to environment dynamics improving stability and reliability. Multiagent system is exploited for microgrid automation through cyber physical system and scaled up using cloud computing concept for larger benefits. Advanced SCADA uses MAS to meet the challenges due to increased penetration of renewable energy resources.

## 8. Conclusion

MAS is implemented in JADE framework for optimal energy management and effective demand side management of a solar microgrid. Solar microgrid model is developed in MATLAB/Simulink platform and it is linked with JADE using MACSimJX for realizing the practical implementation of MAS. A novel control strategy is used for dynamic energy management and fair allocation of resources for demand side management of solar microgrid. In the microgrid, the update rate of the unit dispatch command should be sufficiently fast to follow the unexpected changes of load and nondispatchable generators. So the inherent features of MAS increase switching speed and reduce the network load leading to achievement of lowest possible cost of power generation under intermittent nature of solar power and randomness of load. JADE augments the MAS with its features and further improves the operational efficiency. The proposed framework gives the intelligent consumer the ability to explore all possible logical sequences of options, understand the stochastic environment, and implement the optimal energy management actions to increase operational efficiency autonomously in a distributed environment, bringing MAS closure to real-time implementation. Future work will focus on extension to multiple agents integrating diverse renewable generators (solar and wind) with several intelligent consumers with conflicting requirements. Furthermore, the proposed approach can be implemented in actual microgrids by sending and receiving the environment variables values using Internet Of Things (IOT) technology.

## Figures and Tables

**Table 1:** Device description.

Number	Abbreviations	Description
1	SDP and LDP	Department solar power and load
2	SHP and LHP	Hostel solar power and load
3	BD and BH	Department battery and hostel battery
4	GRD and DSL	Grid and diesel
5	GPR and DPR	Grid and diesel unit price
6	CL and NCL	Critical load and noncritical load
7	LCDP and LCHP	Critical load in department and hostel
8	LNCDP and LNCHP	Noncritical load in department and hostel
9	CA	Control agent
10	SDA and SHA	Department and hostel solar agents
11	LDA and LHA	Department and hostel load agents
12	BDA and BHA	Department and hostel battery agent
13	GA and DA	Grid agent and diesel agent
14	(DSL-LD-CA)	LD and DSL switch controlled by CA
15	(DSL-LH-CA)	LH and DSL switch controlled by CA
16	(GRD-LD-CA)	LD and GRD switch controlled by CA
17	(GRD-LH-CA)	LH and GRD Switch controlled by CA
18	SD-BD	Switch connecting solar and battery in department
19	SD-BH	Switch connecting SDP and battery in hostel
20	SD-GRD	Switch connecting solar department and grid
21	BD-LD	Switch connecting battery and load in department
22	BD-LH	Switch connecting BD and load in hostel
23	SH-BH	Switch connecting solar and battery in hostel
24	SH-BD	Switch connecting solar hostel and BD
25	SH-GRD	Switch connecting solar hostel and Grid
26	SH-LD	Switch connecting solar hostel and LD
27	SD-LH	Switch connecting SD and load in hostel
28	BH-LH	Switch connecting BH and load in hostel
29	BH-LD	Switch connecting BH and load in department
30	DSL-LD	Switch connecting diesel unit and LD
31	DSL-LH	Switch connecting diesel unit and LH
32	GRD-LD	Switch connecting grid and LD
33	GRD-LH	Switch connecting grid and LH

**Table 2:** Case study values (kW).

Time	SDP	LDP	SHP	LHP	DPR	GPR
0	400	200	300	100	10	8
1	200	400	300	100	10	8
2	400	200	100	200	8	10
3	200	400	100	300	8	10
4	200	400	100	300	10	8
5	200	300	300	100	10	8
6	400	200	100	200	8	10
7	200	300	100	200	8	10
8	200	300	100	200	8	10
9	200	400	100	300	10	8
